# Asphyxiation due to obstructive fibrinous tracheal pseudomembrane after closure of repeated tracheostomy in a case of von Recklinghausen disease

**DOI:** 10.1016/j.rmcr.2025.102298

**Published:** 2025-09-26

**Authors:** Taiki Hara, Ken Enda, Taku Maeda, Yohei Ikebe, Hideki Ujiie, Masahiro Onozawa

**Affiliations:** a Clinical Training Center, Hokkaido University Hospital, Sapporo, Japan; b Department of Cancer Pathology, Faculty of Medicine and Graduate School of Medicine, Hokkaido University, Sapporo, Japan; c Department of Plastic and Reconstructive Surgery, Faculty of Medicine and Graduate School of Medicine, Hokkaido University, Sapporo, Japan; d Center for Cause of Death Investigation, Faculty of Medicine, Hokkaido University, Sapporo, Japan

**Keywords:** Asphyxiation, Obstructive fibrinous tracheal pseudomembrane (OFTP), Tracheostomy, von Recklinghausen disease

## Abstract

**Background:**

Obstructive fibrinous tracheal pseudomembrane (OFTP) is a rare but potentially fatal complication, most commonly reported after endotracheal intubation. Its occurrence following tracheostomy closure is poorly recognized.

**Case presentation:**

We report a fatal case of OFTP in a 33-year-old man with von Recklinghausen disease who developed progressive airway obstruction following closure of a repeated tracheostomy. The patient had a history of multiple facial tumor resections requiring repeated tracheostomies due to orofacial deformities. After the eighth tracheostomy and successful tumor debulking, the tracheal cannula was removed on postoperative day 6. At that time, CT revealed only mild tracheal narrowing without intraluminal obstruction, and the patient remained asymptomatic. However, he was found in cardiopulmonary arrest on postoperative day 14. Postmortem CT showed near-complete tracheal obstruction and pulmonary edema. Autopsy confirmed a grayish-white pseudomembrane obstructing the tracheal lumen, with only a 2-mm residual gap. Histopathology revealed fibrinous exudate with neutrophilic infiltration, consistent with OFTP.

**Conclusion:**

This case illustrates that OFTP can occur silently after tracheostomy closure and may lead to sudden death. Vigilant monitoring with CT or bronchoscopy should be considered in high-risk patients with repeated tracheal interventions.

## 1 Introduction

Obstructive fibrinous tracheal pseudomembrane (OFTP) is a rare but potentially fatal complication, most commonly associated with endotracheal intubation. Most reported cases have occurred within days of extubation, and the diagnosis is often made by bronchoscopy. While OFTP is typically linked to endotracheal intubation, the risk following tracheostomy closure is not well recognized. Here, we report a rare and fatal case of OFTP in a patient with von Recklinghausen disease, who developed silent but progressive tracheal obstruction following repeated tracheostomies resulted in sudden death.

## 2 Case presentation

**Fig. 1:**
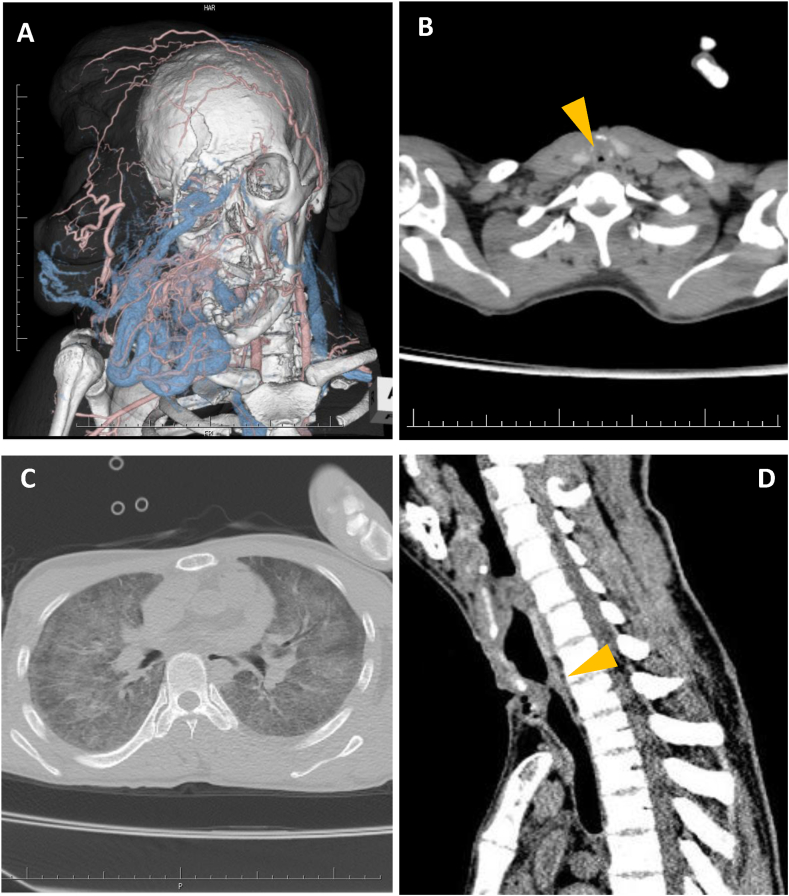
A. 3D CT image showed hypervascular mass that extended to the upper airway, causing severe deformation of the oral cavity and jaw, whereas the lower airway was intact. B. Postmortem CT revealed pinhole tracheal stenosis (arrowhead). C. Postmortem CT showed negative pressure pulmonary edema, suggesting asphyxia as the cause of death. D. Sagittal section of CT showed obstruction locating just beneath of tracheostomy site.

A 33-year-old male was diagnosed with Recklinghausen disease (vRD) at 1 year of age, and he underwent seven surgical tumor debulking procedures between age 18 to 26 to manage progressive facial neurofibromatosis. Due to deformation of the oral cavity and jaw, oral intubation was difficult and tracheostomies and its closures had been repeated for each surgery. At age 33, the facial mass grew again, and the forehead tumor was drooping and blocking his visual field. The facial tumor was hyper vascular mainly supplied by the left superficial temporal artery (Fig. 1A), and embolization was performed via that artery. The patient subsequently underwent mass reduction surgery with an eighth tracheostomy. Mass reduction surgery was successful with resection of a 505-g tumor. A permanent tracheostomy was advised due to the likelihood of future interventions. However, the patient strongly opposed this option, leading to repeated tracheostomy closure. The tracheal cannula was removed on postoperative day 6. At the time, a CT exam revealed only very mild tracheal stenosis with no evidence of intraluminal obstruction within the tracheal lumen. The patient did not complain dyspnea, wheezing or stridor, and remained asymptomatic until postoperative day 13, scheduling discharge within a few days, however the early morning of postoperative day 14, he was found unresponsive in cardiopulmonary arrest in rigor mortis upon the medical team's arrival. Postmortem CT revealed severe tracheal narrowing with pinhole stenosis and negative pressure pulmonary edema (Fig. 1B–D), strongly suggesting asphyxiation as the cause of death.

**Fig. 2:**
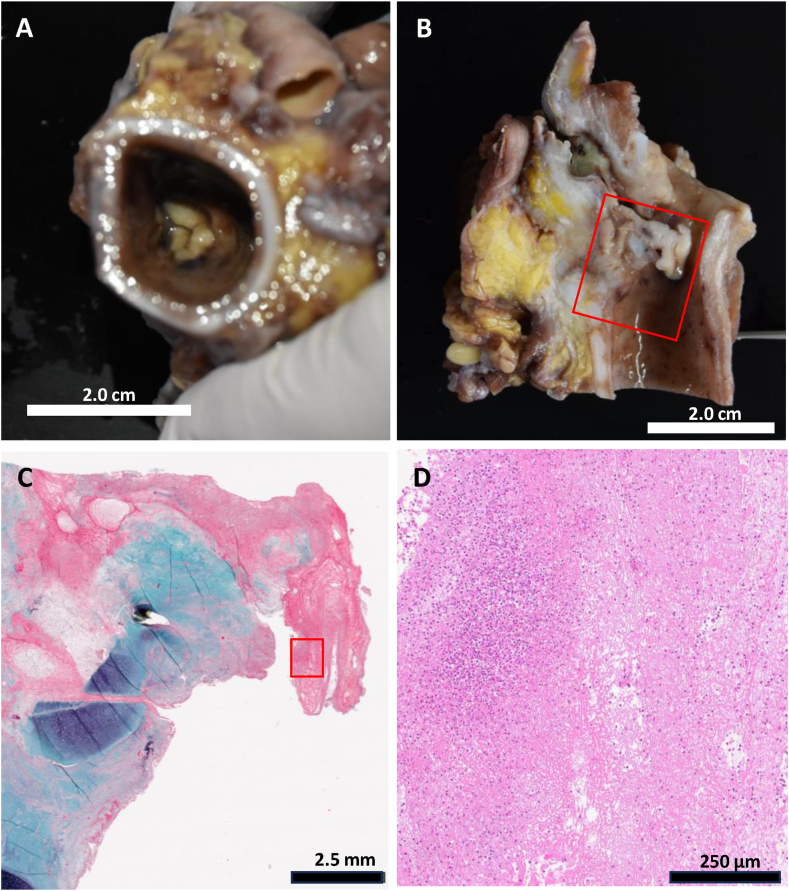
**A.** View from the caudal side of a horizontal section of the trachea showed thick, rubber-like, pseudomembranous tubular necrotic tissue resembling a tracheal valve at the level of the tracheostomy site. **B.** Sagittal section of the trachea shows tissue protruding for 15 mm from the sub-incisional site of tracheostomy into the tracheal lumen, leaving an approximately 2-mm gap on the posterior wall of the tracheal cavity. **C.** The red rectangle area of Fig. 2B is shown. The underlying tissue is composed of areas of exudate, granulation tissue with a rich vascular network, fibrotic tissue with dense collagen fibrosis, and deeper areas with buried accessory glands (Elastica-Masson stain). **D.** The red rectangle area of Fig. 2C is shown in a high power field (Hematoxylin and eosin stain). The tissue is composed of reticulated fibrosis, erythrocytes, and inflammatory cells, mainly neutrophils.

**Fig. 3:**
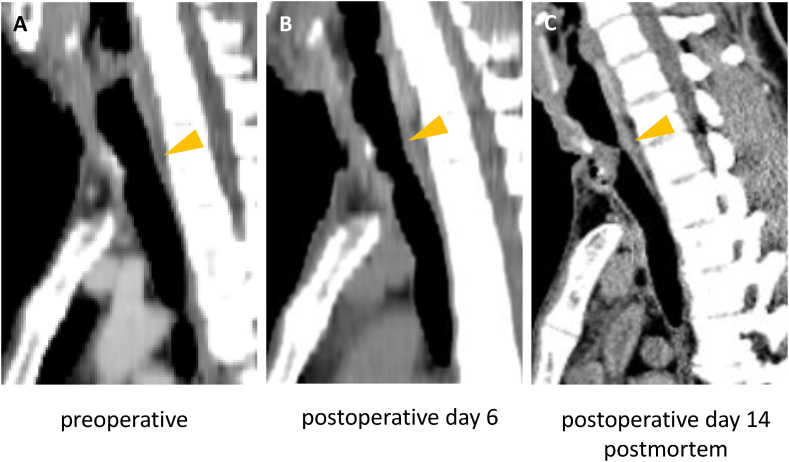
**A-B.** CT images of sagittal section of the trachea. A. preoperation, B. post operation after decannulation of tracheostomy (postoperative day 6), C. postmortem (postoperative day 14). Stenosis of trachea (arrowhead) was observed since preoperation but progressed after decannulation of tracheostomy.

Autopsy confirmed occlusion of the tracheal lumen by grayish-white pseudomembranous tissue (Fig. 2A). A tissue protruded from the sub-incisional tracheostomy site, leaving only a 2-mm posterior airway gap (Fig. 2B). Histopathological examination showed fibrinous reticulation with inflammatory cell infiltration, predominantly neutrophils, along with sac-like exudate trapping (Fig. 2C and D), confirming obstructive fibrinous tracheal pseudomembrane (OFTP). There was no evidence of neurofibromatosis involvement; the lesion consisted only of inflammatory pseudomembranous tissue. Retrospective analysis of CT showed rapid progress of tracheal stenosis between postoperative day6 to day 14(Fig. 3).

## 3 Discussion

OFTP is a rare but potentially fatal complication, the term first proposed by Deslee et al., in 2000 [1]. It is typically associated with endotracheal intubation, with reported symptoms ranging from wheezing and stridor to acute respiratory failure, depending on the degree and location of airway obstruction [[1], [2], [3]]. The first symptoms developed 1 hours to 15 days after extubation [1,4]. Risk factors for OFTP include female sex, tracheal tube cuff pressure >25 cmH_2_O, traumatic intubation, use of a double-lumen tube, prolonged endotracheal intubation, airway stenting [[1], [2], [3], [4], [5], [6], [7]]. Its pathogenesis is believed to involve over-inflated endotracheal tube cuffs, tissue hypoperfusion, and aspiration related injury, leading to abnormal regeneration and formation of obstructive fibrinous membranes [1,6]. Formation of pseudomembrane in our case was just inside of incision of tracheostomy and not located at the tracheal tube cuff site. Repeated tracheostomy and its closure itself seemed to trigger the OFTP in our case.

Pseudomembranes are typically 3–4 cm long, rubbery, and whitish [1,8]. Histologically, they resemble hemorrhagic infarction of the submucosa, with polymorphonuclear cells infiltration and fibrinous exudation. In this case, the presence of reticulated fibrinous fibers and inflammatory cells, particularly neutrophils without specific pathogen, was consistent with previously reported OFTP. Most of the cases with OFTP has been described following tracheal intubation [1], except one case who developed OFTP following repeated tracheal intervention including intubation and following tracheostomy [9]. Although the mild stenosis at the tracheostomy site had been present even before operation, rapid progress of the stenosis occurred after extubation of tracheostomy tube (Fig. 3). Etiology of such a rapid formation of pseudomembrane remains unclear.

OFTP typically presents as upper airway obstruction, with dyspnea and stridor being the main symptoms. However, in some cases, positional dyspnea or exhaustion may prevent the manifestation of stridor, leading to a silent presentation of OFTP [9]. Our patient did not complain of any respiratory symptoms. Given the location of the OFTP lesion, our patient may have experienced positional dyspnea only in the supine position, without symptoms while upright, which could have led to asphyxiation while asleep. Our case reminded the risk of OFTP which potentially result in sudden and fatal acute respiratory failure like previous few cases [1,3].

Although the lesion may develop at the tracheostomy site, its detection is challenging because bronchoscopy through the cannula may not reveal it before extubation. Additionally, post-extubation hypoxia often leads to re-intubation without detailed airway evaluation. Given that OFTP is not widely recognized, its true incidence may be underreported. Symptoms can easily be misdiagnosed as laryngeal edema, bronchial asthma, vocal cord dysfunction, or glottic edema.

While most cases of OFTP were diagnosed by bronchoscopy, a few reports have demonstrated detection of the lesion by CT. [6,10,11]. In our case, CT clearly showed the location and severeness of OFTP. This case underscores the importance of recognizing OFTP as a potential cause of post-tracheostomy respiratory failure and highlights that tracheostomy, like intubation, can induce its formation. Greater awareness and early detection may help prevent similar fatal outcomes. Even if the symptom is mild or absent, screening and follow up by CT would be useful to detect early sign of OFTP for the patient undergone multiple tracheostomy and its closure. This case underscores the need for heightened clinical awareness of OFTP as a potential complication after tracheostomy as well as intubation.

Once OFTP is suspected or visualized by bronchoscopy, prompt interventional management is essential. Reported treatment options include mechanical removal with forceps [5], debulking under rigid or flexible bronchoscopy [1,7], and cryotherapy [9], which may allow safe and effective removal of the obstructive membrane. Early recognition and timely intervention could prevent fatal outcomes, particularly in patients with a history of repeated tracheal procedures.

## 4 Conclusion

This case highlights that OFTP may develop not only after endotracheal intubation but also following tracheostomy closure, even in patients without respiratory symptoms. Because OFTP can progress silently, its diagnosis may be delayed until sudden respiratory failure occurs. In patients with multiple tracheostomies or other high-risk airway interventions, careful follow-up with CT or bronchoscopy should be considered. Greater awareness of this rare entity among clinicians may help facilitate early detection and life-saving intervention.

## CRediT authorship contribution statement

**Taiki Hara:** Writing – original draft, Investigation. **Ken Enda:** Methodology, Investigation. **Taku Maeda:** Supervision, Conceptualization. **Yohei Ikebe:** Methodology, Investigation. **Hideki Ujiie:** Writing – original draft, Supervision. **Masahiro Onozawa:** Writing – review & editing, Visualization, Supervision.

## Consent

Written informed consent for autopsy and publication was obtained from the patients’ parent.

## Funding sources

This research did not receive any specific grant from funding agencies in the public, commercial, or not-for-profit sectors.

## Declaration of competing interest

The authors declare that they have no known competing financial interests or personal relationships that could have appeared to influence the work reported in this paper.
